# World Alliance for Risk Factor Surveillance White Paper on Surveillance and Health Promotion

**DOI:** 10.3934/publichealth.2015.1.10

**Published:** 2015-02-03

**Authors:** Stefano Campostrini, David McQueen, Anne Taylor, Alison Daly

**Affiliations:** 1 Ca' Foscari Graduate School,University Ca' Foscari Venice, S.Sebastiano - Dorsoduro 1686, I-30123 Venice, Italy; 2 Global Consultant, 2418 Midvale Ct; Atlanta 30084; 3 Faculty of Health Science, The University of Adelaide, Adelaide 5005, South Australia; 4 School of Public Health, Curtin University, Kent Street, Perth, 6845, Western Australia

**Keywords:** risk factor surveillance

## Abstract

This is not a research paper on risk factor surveillance. It is an effort by a key group of researchers and practitioners of risk factor surveillance to define the current state of the art and to identify the key issues involved in the current practice of behavioral risk factor surveillance. Those of us who are the principal authors have worked and carried out research in this area for some three decades. As a result of a series of global meetings beginning in 1999 and continuing every two years since then, a collective working group of the International Union of Health Promotion and Education (IUHPE) was formed under the name World Alliance of Risk Factor Surveillance (WARFS). Under this banner the organization sought to write a comprehensive statement on the importance of surveillance to health promotion and public health. This paper, which has been revised and reviewed by established peers in the field, is the result. It provides the reader with a clear summary of the major issues that need to be considered by any and all seeking to carry out behavioral risk factor surveillance.

## 1. Introduction

The World Alliance for Risk Factor Surveillance (WARFS) is an International Union for Health Promotion and Education (IUHPE) Global Working Group founded in 2008, adopting the considerable work of an informal international network of surveillance practitioners and researchers who have met since 1998 in international conferences held in several countries to discuss theoretical and practical aspects of risk factor surveillance. WARFS supports the development of risk factor surveillance (RFS) as a tool for evidence-based public health, acknowledging the importance of this information source to inform, monitor and evaluate disease prevention and health promotion policies, services and interventions. The aims of WARFS are to integrate surveillance as a tool into the mainstream of health promotion; to finalize the definition and conceptual framework of RFS which can be shared and discussed globally; to serve as a reference for researchers, RFS practitioners and countries that are developing RFS; and to share findings, results and experiences with the IUHPE community to facilitate a dialogue regarding the role of RFS.

Our surveillance roots lie in the 1980s with the first experiences of producing measures of the major risk factors linked to NCD (non-communicable diseases). Over time it has moved from an initial epidemiological research approach to a more complex and systematic approach that embeds surveillance in the public health practice offering not only data, but also information for decision-making processes. Even if the main focus remains on risk factors, other information is produced by advanced surveillance systems that more properly could be called surveillance systems for health promotion.

Given this history, WARFS believes it is now timely to define what surveillance in health promotion is and what distinguishes such surveillance. This is not a scientific paper on surveillance, although much of what is written derives from solid scientific research: those interested in more in-depth presentation of the many issues linked with surveillance, here briefly addressed, can find rich information in the international literature to which many of WARFS members have substantially contributed. This white paper is being written to share with a larger audience the knowledge developed so far by WARFS and by its members and to provide a better understanding of the role of surveillance in health promotion and guide any others in the development of a surveillance system. This white paper will also define what WARFS means by surveillance for health promotion and for what it can be used.

## 2. The Theory of surveillance

The word surveillance can be traced to the Latin word *vigilare* from which the term vigil is derived. It derives from the French *surveiller* “to watch over” from the early 19th century. The idea of surveillance has a critical role in public health. In essence, whether termed surveillance, tracking or monitoring, the epidemiological approach of public health has generally implied the observation of changes and trends in the magnitude of factors related to disease in populations over time. This approach provides the background information and intelligence on which public health interventions and program efforts towards disease control and health promotion should be based.

Traditionally, this approach calls for the collection of good quality data providing representative, sensitive and timely results. In recent years, the idea of surveillance has broadened to go beyond the mere collection of data to an evolving concern with analysis, interpretation and dissemination of the data as part of surveillance. Thus one should conceptualize surveillance as a system of related activities ranging from recognizing epidemiological parameters of disease to identifying the public health policies that could influence health and illness. It is the systematic aspect and the dynamic nature of the data collection, analysis and use that separates surveillance from the mere collection of vital statistics and records. A critical point, from our perspective, is that a true surveillance system succeeds only if it fulfills all three perspectives: collection, analysis and dissemination.

**Figure 1.**
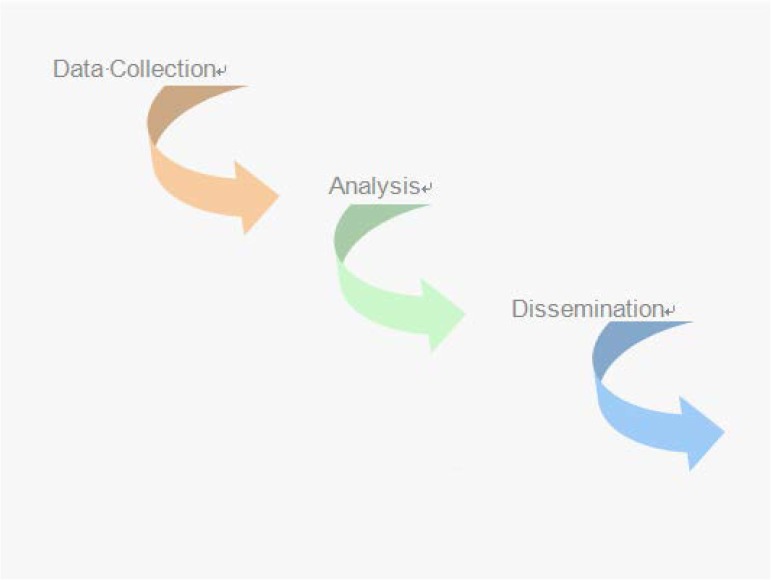
The three aspect of surveillance.

Historically surveillance systems based within the public health epidemiological approach have had two major drawbacks: 1) they have over-invested in data collection and 2) they have lacked a coherent theoretical base. While it is valid to state that public health surveillances systems have in recent years moved to a more comprehensive approach that includes greater emphasis on the analysis and use of the data collected, the lack of a theoretical base that specifies the logic and foundation of surveillance has been less emphasized. In particular, the theoretical underpinning and logic of risk factor surveillance has been a weakness that has led in many cases to a misunderstanding of the approach.

In this WARFS White Paper, we propose to define how we as a working group view risk factor surveillance approaches, describing the theoretical distinctions that we value and pointing out those notions that make our health promotion-based approach distinctive. Our concern is with the ideal and true surveillance system to which we as practitioners in the field aspire. An underlying assumption is that continuous data collection, analysis and application of surveillance provides an evidence base for health promotion and preventive public health. Several other fundamental assertions operate. The system (i.e., data collection, analysis and use) is continuous and population based; the individual (respondent) is not the focus; the social survey is the instrument for the collection of data; time is a critical variable; change over time is central; technical and structural aspects are critical and equitably weighted; and a coherent theoretical base underlies the rationale for the data collected, the analyses undertaken and the use of the information obtained.

## 3. The meaning of continuous in the RFS context

The most contentious issue in the surveillance of socio-behavioral risk factors is the notion of continuous data collection. In an ideal surveillance system - whether the monitoring of the Earth's movement in a seismograph, the use of a surveillance camera in a shop or surveying populations about risk factors - the data collection is continuous. On a regular basis public health practitioners will use the word surveillance to describe data collection survey efforts that are only occasionally in the field collecting. Thus similar-appearing surveys that occur every few months or years will often be described as a surveillance system. Even if systematic, they do not meet the fundamental logical requirement of continuous data collection. The idea of a continuous system also requires that data collection, analysis and use are also occurring continuously. The notion of a continuous system is abstract or ideal. The reality is that, even if one were to collect data every day of the year. it would not be collected 24 hours every day. Practically speaking, data that are collected routinely on a highly regular basis, preferably on a daily basis, with standardized time periods each day can be conceived of as continuous. This would still be an ideal case. The ability to collect data on such a rigorous plan approximating a continuous data stream is conditioned on the instrument and data collection methodology available. In our case, the instrument is the questionnaire-survey and it has limitations, however it is important to note that all instruments have limitations. The shop surveillance camera is actually not collecting images continuously but, rather like a motion picture, recording multiple single images over a fixed unit of time. The seismograph is subject to the random perturbations and movement caused by man-made disturbances occurring in the range of detection. However these two instruments simply reflect types of errors that accrue to the instrument, not flaws in the theoretical basis for the use of the instrument to collect continuous data.

That the instrument for data collection is the survey is a critical point that is often not considered theoretically because it is simply assumed to be the basis for risk factor surveillance. In fact, the survey approach is rather recent and highly tied to the social sciences, notably sociology, and was considerably refined by the rise of computing devices able to handle large data sets. Fundamentally a social survey is not a method to investigate single individuals; it is theoretically tied to a population statistics approach. Many assume that the instrument is the questionnaire that is used in a survey, but it is more appropriate to conceive of the whole survey as the instrument. Thus the survey is a structure that houses components that are questions based on variables that relate to socio-behavioral concepts that relate to health. The theory underlying the survey is very complex, based on a modeling approach that assumes that through a patterned number of questions, the socio-behavioral patterns of populations of respondents can be modeled. Once modeled the results are entered into a population frame as an entity. The result of many such models aggregated at a point in time reveals a static picture of social behavior in the population. Like all instruments, the survey has many possibilities for it not to work well and those practicing the art of surveillance spend a great amount of time trying to get the instrument to work as well as possible. Those who work with surveys refer to such problems with terms such as reliability, validity, measurement error and others. However, as with all instruments, when allowed to run over a long period of time, the errors become understood and therefore less problematic. This is one of the most important points of continuous data collection - as the instrument (the survey) is used day in and day out, its operating characteristics, positive and negative, become better understood by the operators of the system. This is a unique aspect of true surveillance; it is a learning system. The same cannot be said for the occasional population survey.

## 4. The meaning of population-based in the RFS context

Both the social survey and surveillance are rooted in a population-based, statistical approach. That is they yield results about the characteristics of the population in which individuals are actors. It is a common error to assume that the social survey tells one about an individual in a population when in fact the individual, the respondent, is in actuality merely one informant on behaviors in the population and at the same time an informant on the social structural characteristics of the population. Thus in a socio-behavioral risk factor surveillance system, one is not monitoring changes in individuals but changes in behaviors in the population. Perhaps because of the initial epidemiological, individual, medically-based orientation of many who work in public health and use surveillance data, this remains a difficult conceptual problem. In considering social surveys on economic and labor type variables and their change over time, we easily attribute the results from a population perspective and do not seem to be driven to anthropomorphisms.

## 5. Time and change as theoretically central concepts

Given the central notion of socio-behavioral risk factor surveillance of tracking changes in behaviors in the population over time, as public health practitioners, we are interested in both the magnitude of a behavior and its change over time. Measuring both the magnitude and the change in behaviour at the same time is challenging. Theoretically, this aspect is analogous to the Heisenberg uncertainty principle that states that certain pairs of physical properties, like position and momentum, cannot both be known with precision. That is, the more precisely one property is known, the less precisely the other can be known. Thus if one wants to know the precise percentage of smokers in a population at a given point in time, then a census of the population must be undertaken; if one wants a close estimate, a large sample survey would suffice. However, the exact percentage of smokers in a population is highly variable over time and if you want to understand that variation over time, you have to conduct surveillance. Obviously, to use the smoking example, there are many reasons, defined by social scientists as variables, why smoking may change in a population over time, including demographic changes, deaths, illness, taxation, economic downturns, custom, popularity, to name just a few. It is quite clear, given all the changing influences, that it is very unlikely that the percentage of smokers would be the same at any two points in time over a period of months or years. For public health policy, the concern is mostly on increases and decreases of smoking behaviors. The reality in surveillance is that the all the population characteristics as well as the behaviors measured by the instrument are changing over time, some more profoundly than others.

It is clear that the reality of surveillance introduces high complexity and that is one of the theoretical characteristics of surveillance that distinguishes it from other approaches to data collection and presents unique challenges to data analysis and interpretation. While many data collection approaches, (e.g., surveys, registries) tend to move towards data analyses that are reductionist, surveillance data cannot so easily be reduced to single variable analyses. The reality of surveillance is that all the measured variables are changing over time in some observable relationship to each other. Thus, any analytic approach must be very dynamic. Fortunately, modern statistical approaches exist to address that dynamic.

## 6. Other implied theories in surveillance

While we have explored some underlying theoretical dimensions of surveillance, there are many that remain. Implicit in the idea of socio-behavioral changes over time are many assumptions about the causes of these changes. Herein one enters the world of ideas and theories about individual and societal change over time. One may classify these theories of causality into what can be termed little theories, mainly at the individual behavioral level and big theories that operate at the societal level. Among the former are ideas, such as risk behaviors, lifestyle concepts, personal behaviors and many others, which stem from a rich socio-psychological literature on human behavior. Among the big theories are broad concepts such as economic, globalization, urbanization, religion and other broad societal perspectives that changes in the population over time. The issue with such theories for those engaged in surveillance is how to incorporate these ideas into the instrument of surveillance and lacking the ability to do that in some cases, how to analyze the data we have in a way that incorporates theoretical explanations from these many causes.

To give a concrete example, take the phenomenon of obesity whose trajectory in the West has been so well documented by extant surveillance systems, such as the well-established USA-CDC-based BRFSS (http://www.cdc.gov/brfss/index.htm). Clearly, there has been a proportionate change in the weight of the population over time, but what are the causes? Some of the answer must be in the data that have been collected, and in many cases not discovered because of lack of analyses, a point that leads us from theory to methods.

## 7. Methods of RFS data collection

To meet the theoretical parameters of a surveillance system as outlined above, a surveillance system must have a proper methodological base. Surveillance needs appropriate methods for data collection (to ensure continuity of the data stream), for analysis (to find evidence of change, trends and evolutionary processes) and for reporting (to link surveillance, and to support policies and public health actions).

From a statistical point of view, surveillance is characterized as a system of data collection and analysis based on repeated surveys with independent samples from the same population. Continuity in the data collection is critical for the surveillance system purposes, so that the data can be used to analyze trends and changes, to inform about emergencies and health outbreaks, and to allow space and time aggregation for better and more useful estimates. The continuity of the data collection, as differentiated from other systems based on surveys repeated at greater intervals of time (if not without re-defined periodicity), offers specific peculiarities which are particularly interesting for surveillance purposes. The availability of a continuous data stream allows for two distinctive strategies: aggregation over time––to get a bigger sample size which is useful for increasing the precision of estimates, particularly at the local level; and aggregation over space to allow for more sufficiently precise point-in-time observations.

In an ideal surveillance system, in which data are collected continuously, data could be analyzed over large periods of time (one, two or three years) enabling analyses to be undertaken at the local level, while national or regional samples could be pooled together when the interest is in the evolutionary process rather than geographic area. For these analyses the more observational points the better, so an analysis by month or by week is more informative than one at less frequent intervals.

As previously mentioned, continuity is an important aspect of risk factor surveillance. Moving from the theoretical to the practical definition of continuity can vary with the needs of the surveillance system itself. Thus the system could be reasonably different from country to country. From a methodological point of view, the definition of continuity in a surveillance system cannot be physical or mathematical but only logical. The data collection in a surveillance system should be sufficiently continuous so as to be capable of catching and monitoring the trends and changes of interest for the public health system to which the surveillance system is linked. As some variables are very stable over time (e.g., prevalence of asthma, diabetes), and others may vary very rapidly (e.g., fruit and vegetable consumption after a major health promotion campaign), the interval of data collection, and therefore the definition of continuity will also depend on what are the key variables of interest for the system.

It should also be remembered that most variables need a minimum of monthly observation to consider seasonality effects or to detect the effect of an intervention for evaluation purpose. Research on sustainability shows that a system that is continuously running is more cost efficient than a survey repeated every 2–3 years, and more observations over time mean more information on the dynamic of the phenomena under surveillance.

To be most effective in serving these needs, and to be an effective public health tool, there are desirable qualities for a surveillance system which include 1) simplicity -the system needs to be structured in a simple and straightforward way so that it is easy to maintain, update and use; 2) flexibility –the surveillance system needs to be flexible enough to address any emerging public health issue the countries may face and easy to change by adding information, or changing the target population; 3) timeliness –data from any surveillance system should be released in a timely manner whenever the information is needed, and information must be relevant and within budget; 4) reliability –notwithstanding the need for timeliness and flexibility, reliability is also very important in an endeavor to detect all chronic disease and risk factors of interest within the relevant population; 5) acceptability –for a system to be acceptable, it is important that it is acceptable to all concerned; 6) utility –the system needs to be perceived to be good value for the organization; and 7) sustainability –related to the concept of utility is the necessity to have a sustainable system so that the trends for which it is designed can be generated, and this means that the system must resourced over time.

A surveillance system must have an ongoing long-term plan. Ongoing data are important for generating accurate time trends to provide early warning and for developing and evaluating programs and interventions. Many surveillance systems have been created only to be dropped a few years later during a change in government or manager, resulting in wasted resources. Other surveillance systems change their datasets annually including the type and definition of variables which leads to inconsistent data over time. There is a careful management trade-off between stability of instrument and flexibility.

Methodologically, a systematic surveillance system is desirable but still often difficult to achieve. Systematic means that there should be a consistent set of core questions, the questions should be determined via a systematic process, there should be standards regarding adding or deleting questions, and there should be clear and definable definitions around the variables. In addition, the system should be directly linked to public health practice and interventions. Also important is the systematic linking of the data to dissemination.

## 8. Methodological process and questionnaire construction

The importance of developing an appropriate questionnaire is well known in any social research field, as are the issues related to its construction such as question wording and question order. The surveillance questionnaire design needs close consideration to enable the collection of accurate information to: meet the needs of potential data users (especially policy needs) in a timely manner; facilitate the work of data collection, data processing and tabulation; ensure economy in data gathering by avoiding collection of any non-essential information; and permit comprehensive and meaningful analysis and utilization of the data collected. Besides these concerns, surveillance presents unique challenges for questionnaires. By the very definition of a surveillance system with its feedback component, the questionnaire cannot remain the same over time but at the same time, has to have some consistency to allow the observation of trends over time. To achieve this, most of the existing continuous data collection surveillance systems use a stable core set of questions and handle necessary changes to these by using algorithms.

The stable core part of the questionnaire is essential for a successful surveillance system but even within this, there is flexibility. The surveillance system can be designed to include certain questions every month/year (i.e., a fixed core), others in alternate years/months (i.e., a rotating core) and still other questions introduced as emerging core for newly arising urgent topics. A surveillance system can also include optional, standardized sets of questions regarding specific topics either as core or as optional modules.

Where possible, a surveillance questionnaire construction should include questions that are also being asked in other relevant states/countries so that country and international comparisons can be made. This increases the utility of the system and contributes to its sustainability.

## 9. Methodological process and sampling strategy

While surveillance does not require new sampling theory or sampling frames from other survey methodologies, choosing one based on random sampling is essential. Sampling design for a surveillance system may vary from one country to another depending on the needs and resources available. The only requirements are that the samples are representative of the underlying population (the subject of surveillance); that the probability of selection is known; and the sampling strategy is based on scientific principles. These conditions are necessary to both properly weight the data, if required, and conduct appropriate analysis. It is a good principle to seek advice from sampling professionals to ensure these conditions are met.

### 9.1. Data Collection

Data collection is typically the core activity of a surveillance system –its hardware. The collection needs to be regular, sustained over time and relevant to policy makers and responsive to the political background. The issues of surveillance data collection are those of any data collection.

### 9.2. The timing of the data collection

As time is the most important aspect of surveillance data collection decisions about the study period (month, quarter, and year), the timing (day and evening hours) and days (all or most of the 7 days in the week) of interviewing need to be considered carefully within the context of the purpose to which the data will be used. The principle is, within the constraints of the resourcing, the more often the better.

### 9.3. Method of data collection

Interviews can be conducted either directly by the organization or outsourced. In many countries, interviews are conducted by using computer-assisted telephone interview systems (CATI). In countries where a telephone survey is not the appropriate mechanism to conduct surveillance, there are other alternatives such as use of face-to-face, web-based, mail questionnaires and mixed mode surveying. Each method has its own challenges for surveillance. These issues are debated within the published literature and research on them is an ongoing priority for those engaged in designing and maintaining good quality surveillance data collection systems. The use of multiple modes of data collection is increasing as are the development of new sampling frames. Methods of data collection should include consideration of alternative, cost-effective ways to ensure longevity of the system, taking into account the popularity of the cell/mobile telephone, and the difficulties associated with mixed mode surveying.

### 9.4. Training and monitoring of interviewers

This is an essential component of data quality. Well-constructed and easy to follow procedure and process manuals should be available and normal quality assurance activities for survey data collection followed. In surveillance, training and monitoring of interviewers is an essential component of data quality. Well-constructed and easy to follow procedure and process manuals should be available and normal quality assurance activities for survey data collection followed. The feedback loops of a surveillance system can only work well if the data collected has integrity and is as valid and reliable as possible given the collection method. Ideally, surveillance systems contain within themselves feedback mechanisms to ensure quality. An internal evaluation process that includes a data-quality-control report and internally sets up checks and balances, or external audits should be part of the system. The great advantage of surveillance is that it is possible to sustain a cadre of interviewers over a long term.

## 10. Data Management and Processing

Modern surveillance has greatly benefited from the available electronic resources found globally today, making the maintenance of data sets reliable and acceptable. Data processing is an integral part of any surveillance system. Keeping the data cleaned and ready for use as soon as possible so that the data remains as timely as possible, requires a well-documented and run maintenance system. In processing data, weighting may be used to make the sample a better representation of the underlying population. Pre- and post-stratification weights can be used to try to correct for any bias associated with the method of data collection (such as households without telephones in the case of a CATI survey) and characteristics of the response population (e.g., young males tend to be least likely to respond to surveys). Pre-stratification can help with ensuring adequate numbers of sub groups are surveyed if these groups form an important part of the organizational reporting structure (this also helps with sustainability and utility). Post-stratification can be used to adjust for under-representation of identified demographic groups, with age and gender being the most common adjustment. Newer advances in weighting known as raking are opening possibilities for better population representation when estimates or prevalence are required.

Table 1 shows the possible information that can be produced with various types of surveillance activity. The analyses conducted determine what information can be generated from a dataset, which in turn determines to what use that information can be put. The table clearly shows that an ongoing data collection, once established, offers the widest variety of analytical possibilities and the most flexible use of the data to generate information. The uses of a good quality continuous data collection are many. They can and should make powerful contributions to the health promotion world. Not only can the information be used to inform, support and guide program development, it can be used to evaluate it on three levels: immediate, medium term and long term impact. It can be used to detect times when a campaign works best; the optimal time to rerun a campaign; and identify the people on whom it works as well as those on whom it does not work. It can be used as a baseline measure for all major socio-behavioral and health conditions and to predict potential health promotion, intervention and service use needs. Changes, trends, spatial and geographical differences must be evaluated with proper statistical tools that take into account the peculiarities of the surveillance data.

Surveillance is a valuable tool for public health but the information extracted from a surveillance system is only as good as the data it is based on. Maintaining the highest quality in sampling, collection, maintenance and weighting will ensure that the information from the system is relevant, reliable and robust. Having systems in place to ensure these activities are routinely evaluated will assist these processes and enable timeliness to underpin the provision of the information.

The principles outlined in this section should underpin any surveillance data collection method.

## 11. Use of surveillance data

Surveillance systems address different needs such as estimating the magnitude of a problem, determining the geographic distribution of illness, portraying the natural history of a disease, detecting epidemics, defining a problem, generating hypotheses, stimulating research, evaluating control measures, monitoring changes in disease, detecting changes in health practices, tracking values, and facilitating planning. They can also provide data on health risk behaviors associated with the leading causes of premature mortality among adults as well as clinical preventive health practices, and health-care access related to chronic disease and injury. Data from surveillance systems can track health objectives, plan and evaluate health programs, implement multiple disease-prevention activities and support health-related legislative efforts.

A surveillance system can provide population-based information on a range of physical, social, economic and cultural factors relevant to health and associate these with the effects of health promotion campaigns and interventions. The strengths of an evidence base which includes surveillance of key factors associated with health and wellbeing include transparency and accountability in the practice of health promotion, the quantifiable demonstration of efficacy and effectiveness (encompassing a broad definition of surveillance) and the identification of successes, gaps and early-warning signs of new and emerging issues.

The first and most obvious challenge for a surveillance system is that its content must contain the relevant data to enable identification and description of factors leading to good or poor health in the population; the second is that the sampling and methods of collection must enable the production of reliable, robust data to support appropriate analysis and reporting of these key elements; and the third is that the information must be used by those who make policy and plans and by those who implement these. To date, the focus has been on the first two challenges with many high quality papers and discussion produced. However, the third area, the use of the data, has been less well researched and reported, partially because many of the uses are internal to health systems, but also because the use of the information is dependent on the type of analysis techniques that are used, many of which are as germane but less used in health promotion.

**Table 1.** Data uses by frequency of collection.

**Data uses**	**Frequency of data collection**		
	Rare(5–10 years)	Bi-annually/Annually	Continuous
1. Point prevalence	✓	✓	✓
2.Period prevalence	✗	✗	✓
3.Description of at risk populations	✓	✓	✓
4.Trends over time with multiple comparison points after initial five year collection period	✗	✗	✓
5.As part of a pooled dataset	✓	✓	✓
6.As part of a meta-analysis	✓	✓	✓
7.Recruitment for further research	✓	✓	✓
8.Recruitment of controls	✓	✓	✓
9.Sample selection for future studies	✗	✗	✓
10. Interrupted time series for evaluation of change due to an event	✗	✗	✓
11. Identify and quantify the effect of seasonal variation	✗	✗	✓
12. Identify the effect of time per se within the context of local conditions	✗	✗	✓
13. Model building to identify associations	✓	✓	✓
14. Factor identification associated with an event	✗	✗	✓

Among surveillance system practitioners, there is the recognition that the use of the data will dictate not only how the data are collected but also the analyses that can be used on those data. What is not discussed in any great detail in reports, and is often omitted completely, is how the information produced will be used. More often there is a small section on how the information could possibly be used in broad brush terms such as to inform policy but little effort is made to transform the data into the type of information that is practical and easy to use in policy, planning and evaluation. This is recognized as the basic problem of knowledge synthesis and translation.

The breadth of opportunities for the use of surveillance data is associated with the frequency of collection and questionnaire content. The recognition of the importance of time has a concomitant outcome in that it can only be used to its full potential if the data collection period is very frequent, preferably continuous.

## 12. How surveillance contributes to health promotion and education

It is our contention that no evidence-based public health program can function well without some form of socio-behavioral surveillance system. A recognized goal of health promotion and education programs, campaigns and interventions is to encourage a change in behavior and/or to maintain a behavior that is conducive to good health and wellbeing. Another goal is to stop or at least delay the onset of disease, particularly long term chronic conditions. Broadly speaking, there are several areas of use for surveillance data to assist this goal, some of which can only be done with continuous collection.

## 13. Surveillance data can be used to investigate changes in behavior in relation to a health promotion/education intervention

Commonly, health promotion/education interventions are designed to reach a target population over a period of time and using a range of strategies. These campaigns or interventions tend to be evaluated by a monitoring system over the period of the campaign that asks for recognition of the campaign/education badging and/or slogans and recall of the message. A continuous surveillance system can show what happens to health status throughout the year and across years with special reference to the times the campaign is running. While causal attribution cannot be made, an association and the strength of that association can be shown.

## 14. Surveillance data can be used to evaluate the long-term effects on health status and health promotion programs

Closely related to the first use of surveillance data is the longer term evaluation of the effectiveness of a health promotion campaign. It is widely acknowledged that even very successful health promotion campaigns often take quite a long time to have real impact on behavior, however the size of the effect, when it occurs and how long it lasts are rarely, if ever, measured. Surveillance data can be used to provide that information. These should be key pieces of information for evaluating the effectiveness of the campaign and these can only be estimated when the data has been consistently and continuously collected over time.

## 15. Surveillance data can be used to predict future trends, health resource use and newly emerging health issues

Continuous data collection allows for many time measures and as a consequence produces more accurate assessment of present trends and more reliable predictions of future trends than the current systems. Continuous systems can also offer early identification of high resource use periods; identification of the best time to introduce an intervention; what effect an intervention has; finding the optimal time between the end of one intervention and the start of another for maximum effect; identification of the time(s) when the majority of the effect occurs (and subsequently the most efficient use of resources), and conversely identifying when the intervention is no longer effective; and identification of what forms of the intervention were most effective in relation to seasons/times of the year. This breadth and detail of information can be used to produce more effective, efficient and efficacious interventions and campaigns.

Continuous data collection systems offer the possibility of identifying emerging health issues at an early stage and with that, the potential to intervene before the issue escalates. A good example of health professionals missing a key change in health behaviors was the failure to recognize and act on the changing weight pattern throughout the world. The obesity issue is relevant for all developed countries where the issue has progressed from a problem to a crisis before it was recognized as a priority for action. As a result, immediate and costly interventions are now required not only to contain the crisis but also to cater for the consequences of the crisis in terms of the associated health outcomes, provision and utilization of health care, and the resourcing and provision of the necessary bariatric equipment and treatment services.

## 16. The link between existing methods, analyses and uses of surveillance systems

All socio-behavioral surveillance data collections, as long as they are regular and frequent, offer the opportunity for the exploration of health-related associations that can be used to inform and support new and emerging concepts for program development. The more regular the collection the greater the opportunity for this to occur, as there is more information contained within the collection, including time. Some of the research-related uses are described below.

## 17. Surveillance data can be used to provide links between information about socio-political conditions related to time and place, and their association with health outcomes and socio-behavioral risk factors

This is a powerful tool for looking at socio-political conditions at any particular time and examining their longer term effect on health-related outcomes over a number of geographic levels (local, national and global). It offers the ability to place information within the context of time and place within a social setting, country or condition.

As long as there is a linking variable, any socio-behavioral surveillance data is capable of being attached to another dataset. The most common linkage point is geographic location which can be at a high level, such as suburb or statistical division, or at the individual level if the information for individual geo-coding is available on the dataset.

This offers the opportunity to link with other existing information about people and places. The scope to enhance the understanding of surveillance data based on place is limited only by the information that is available about it, and collaboration between agencies that collect and keep this information will be essential to ensuring that the data are valid, useful and appropriate.

The increased ability to improve the planning and design of physical spaces and to develop strategies appropriate to the socio-political environment, based on these linked data systems, is immense and could have a significant effect on health promotion and interventions, as well as health service planning and provision.

Socio-behavioral surveillance data of any kind that collects permission and information from the respondent can be used to provide a link with which to add information from administrative health data collection systems, thereby providing a quasi-cohort database for examination of an individual before and after the data collection period. The potential to identify the time between the appearance of a socio-behavioral risk factor and the appearance in the health system with a related health condition has not yet been explored.

## 18. Surveillance data used to add context to time-series analysis examining a specific event/change in time

The continuous data collection also offers the ability to provide context within a more specified domain, such as a health promotion campaign, a legislative change or other event through the use of sophisticated analytical techniques utilizing blocks of time. The addition of this valuable context enables the health promotion/education professional to evaluate where their interventions were successful and with whom, as well as identifying those who did not change, whether was the target was reached or whether an unintentional audience was reached.

## 19. Surveillance data used to model socio-behavioral health determinants

Having an up-to-date and evolving continuous data collection system enables the construction of models to identify socio-behavioral determinants of health and how they are interacting, that is whether they appear causal, modifying or intervening. These data can also be used in the investigation of emerging and important new determinants of health whether causal, modifying or intervening. This is a relatively unexplored but potentially powerful use of continuous systems but is obviously dependent on a continuous feedback system to allow for the modification or addition of questions to meet emerging trends, changing conditions or new information from the research world. The ability of the continuous data collection system to respond to these in a timely and effective manner is one of its strengths.

## 20. Ongoing issues for surveillance systems

There are challenges ahead for surveillance systems if they are to fulfill their potential as timely, flexible, relevant sources of information for policy development and evaluation. In order to ensure that surveillance systems reach their potential, the issues considered below need to be addressed as a matter of priority. These priority areas are critical for those taking a health promotion perspective of surveillance.

## 21. Translating surveillance information for policy makers

Policy makers and program developers often assert that the information they need is not available in the form(s) they want it. Many do not understand the population-based approach to information and those that do, may not understand how best to present or use it. There are two important ways to approach this problem: 1) to have more surveillance practitioners involved in the development of policies and programs; and 2) to train people to become information brokers who can bridge the highly technical world of the surveillance system and the world of information integration, synthesis and presentation for use in policy and programs. These two approaches do not have to be mutually exclusive and it may well be that both together would provide the best and most complete use of surveillance based information.

## 22. Surveillance systems call to action

Surveillance systems can be used to inform in the passive sense, that is as a series of articles or reports, but they can also be used as a call to action. This latter use is not well developed but is arguably the most important use of a surveillance system. Innovative ways to communicate the call to action for the public have been developed and are in use in some countries such as Canada and the United States. All RFS need to incorporate similar innovations.

## 23. Surveillance systems must be flexible and consistent at the same time

The importance of consistency in surveillance data collections is paramount as trend identification and prediction is impossible without it. It is vital to the viability, validity, reliability and usefulness of a surveillance system that the trends, predictions and interpretations are based on data that reflects as accurately as possible the elements of the health-related behavior, status, association or outcome. However, the very consistency that is necessary for good trend analysis can also be a trap in two ways. In terms of what is collected, if the wrong questions are asked or if the right questions are asked in the wrong way, information may be produced that is biased, inaccurate or misleading.

The desire for consistency can also interfere with the need to be responsive to such issues. This becomes a problem if the information being collected is no longer valid or relevant. An important component of surveillance systems is the capacity to be responsive to changing demands arising from a variety of sources. Surveillance systems based on continuous collection make it easy to accommodate the introduction of questions to examine the effect of an unexpected disease outbreak, an unanticipated event or an impromptu legislative change. The capacity of surveillance systems to be both consistent and flexible is unique, providing information to policy and programs that is both reliable and responsive.

## 24. Privacy

While surveillance is not a focus on the individual taking part in the data collection, many believe there is value in linking surveillance data with other personal data sets thereby adding considerable explanatory power to the combined data set. With the availability of rapidly improving technology, the potential for sophisticated data linkage and analysis has become more apparent. Yet these same advances have resulted in greater concern about privacy and tightening of privacy standards for most research. This is an area that requires more effort by those in public health and health promotion. The aim of linking surveillance data with administrative and other datasets is to ensure that information about socio-behavioral risk factors are as useful and informative as possible without violating the privacy of the respondents. This will be important to the ongoing viability of socio-behavioral risk factor surveillance and vital to data linkage potential. What constitutes a functionally de-identified database for a surveillance system to be linked with private data should be resolved as a matter of priority.

## 25. Sustainability

Unless data is collected frequently, consistently and using the best survey techniques, the information required by health promotion/education programs will be at worst, non-existent and at best, sporadic and of limited use. The key to surveillance is the regularity of data collection and this requires consistent resourcing over time. The case needs to be made for this, particularly when health systems are competing for funds within themselves, not to mention across other government agencies. Data collections, relatively speaking, are not prohibitively expensive and continuous systems are no more costly than point-in-time annual surveys. The argument for data collection should be easy to make providing the information from the system is timely, relevant, useful, and easy to obtain and use. This is the challenge for all data collection systems. Without that, sustainability will always be a problem.

If information from data collections systems becomes essential for business by being embedded in operational or strategic plans, sustainability is ensured. The only way that this can happen is if the information provided is ready for use at the time required. Continuous collections offer the best potential for this to occur but any system can be designed or modified to ensure the ability to deliver the information as and when required.

## 26. Technical challenges

With the increasing numbers of mobile/cell phones available and the increasing options for communicating without landlines or their equivalent, the challenge to survey systems based on telephone interviews becomes greater. New and innovative ways to reach populations in a timely manner are required. Better knowledge of what people use to communicate and how best to access them for surveys are now necessary. They should be based on solid research providing answers to the questions: what is the present/projected case? who is affected? how does this affect estimates of population based health? and what are the effects of the different methods available on these estimates? Key areas to be considered include population coverage; timeliness of both collection and reporting; accuracy and reliability of estimates; knowledge of limitations of these estimates and effects of different methods on the information produced.

## 27. A skilled workforce

Hand in hand with the technical issues of data collection are the knowledge and skills of the surveillance planners and the data analysts. As discussed previously, if the questions asked are not right, then no amount of clever analysis will overcome this. To ensure that the surveillance activity is accurately monitoring the population, a body of planners is required. These need to know what the present situation is, how it has changed, what might be likely to happen and how they can best monitor the environment to provide those answers. This is a highly skilled occupation as the breadth of knowledge required is extensive and the ability to assess and respond to technical information also requires a broad knowledge base.

Provided the data collected is good surveillance, then the analysis of those data needs to be as accurate and as informative as possible. Knowledge of the known and reliable survey analysis techniques needs to be supplemented with knowledge of the new emerging innovative techniques. This may come from another area such as the clinical research body. The provision of training for analysts of surveillance data is essential if the system is to be able to produce the information that is potentially available and which can be most useful.

## 28. Surveillance in economically developing countries

NCDs have become the first cause of mortality and morbidity throughout the world, regardless of the level of economic development in individual countries. It is common sense to see public health surveillance as an approach for all countries regardless of economic development and the level of the health system. The useful information that surveillance can offer is vital for any country and any public health system. The importance and the role of surveillance cannot be limited by how it is conducted and alternative innovative data collection for all levels of economic development should be a matter of priority. Arguably the limited availability of resources could be an additional reason to run a surveillance system that could reasonably offer useful information about where and how these resources should be applied.

